# Menstruation and menopause in autistic adults: Periods of importance?

**DOI:** 10.1177/13623613211059721

**Published:** 2021-11-26

**Authors:** Annabeth P Groenman, Carolien Torenvliet, Tulsi A Radhoe, Joost A Agelink van Rentergem, Hilde M Geurts

**Affiliations:** University of Amsterdam, The Netherlands

**Keywords:** adults, autism spectrum disorders, menopause, menstruation, women

## Abstract

Autism spectrum conditions were once seen as a predominantly male condition. This has caused a paucity of information on common events in the lives of women, such as menstruation and menopause. Some smaller studies indicate that autistic women might suffer from increased difficulties surrounding these events. This study aims to investigate whether autistic women experience more frequent premenstrual dysphoric disorder, and increased complaints surrounding menopause. In partly overlapping samples (premenstrual dysphoric disorder, *n* = 70, *n*_ASC_ = 28, *n*_comparisons_ = 42; menopause, *n* = 65, *n*_ASC_ = 30, *n*_comparisons_ = 35), we investigated premenstrual dysphoric disorder prevalence and menopausal complaints. In 70 individuals, we did not find an increased prevalence of premenstrual dysphoric disorder in autistic women (14.3%) compared with non-autistic women (9.5%). In 65 women aged 40 years and above, we found that autistic women did experience higher levels of menopausal complaints. In autistic women, higher menopausal complaints were associated with higher levels of depression and autistic traits. In non-autistic women, menopausal complaints were associated with increased inattention, hyperactivity/impulsivity (i.e. attention deficit hyperactivity disorder traits), and depression. With this work, we show the important role that major reproductive milestones can have in an autistic woman’s life.

**Lay abstract:**

Autism spectrum conditions were once seen as a predominantly male condition, but this has caused research to have little focus on women. Therefore, little is known about menstruation and menopause in autism spectrum conditions. Some smaller studies indicate that autistic individuals might suffer from increased difficulties surrounding these events. This study aimed to investigate whether autistic women experience more frequent premenstrual dysphoric disorder, causing extreme physical, emotional, and functional impairment. In a partly overlapping sample, we also examined whether women with autism spectrum condition experience increased complaints surrounding menopause. We did not find an increased prevalence of premenstrual dysphoric disorder in autism spectrum conditions (14.3%) compared with non-autistic women (9.5%). Those with autism spectrum conditions did experience increased menopausal complaints. These menopausal complaints were associated with higher levels of depression and autistic traits. In non-autistic women, menopausal complaints were associated with increased inattention, hyperactivity/impulsivity (i.e. attention deficit hyperactivity disorder traits), and depression. With this work, we show the important role that major reproductive milestones can have in an autistic woman’s life.

As autism spectrum conditions (ASC) were once been seen as a predominantly male condition, far fewer studies have focused on ASC in women^1^ (Loomes et al., 2017). Moreover, as the classification itself is only now reaching adulthood, there is a shortage of information about autism in adulthood in general (Lai & Baron-Cohen, 2015). Although we know that major reproductive milestones (e.g. menstruation and menopause) can have major effects on the lives of women (Schneider & Birkhäuser, 2017; Schoep et al., 2019), currently few studies focus on how these affect autistic women,^2^ even though there is some indication that autistic women are differently affected by these hormonal changes (Moseley et al., 2020, 2021). As this could indicate a differential need in autistic and non-autistic women, it is of the greatest importance to address this knowledge gap, by mending this imbalance in research.

During the late luteal phase, in the week before menstruation, around 5%–8% of women (Cohen et al., 2002; Wittchen et al., 2002) experience debilitating emotional and physical symptoms, combined with functional impairment, also called premenstrual dysphoric disorder (PMDD) (*Diagnostic and Statistical Manual of Mental Disorders* (5th ed.; DSM-5; American Psychiatric Association, 2013)). PMDD is often comorbid with mood and anxiety disorders (Wittchen et al., 2002), but also associations with less expected comorbidities are reported, (Fernández et al., 2018; Kepple et al., 2016) or schizophrenia (Ullah et al., 2020). Also in ASC, there is evidence of a higher prevalence of PMDD. Lever and Geurts (2016) reported that almost 21% of autistic women—compared with 3% of non-autistic women—suffered from PMDD. A much more worrying percentage is reported by Obaydi and Puri (2008), where it was found that 92% of autistic women with learning disabilities experienced late luteal phase dysphoric disorder (an earlier description of PMDD) compared with 11% of non-autistic women with learning disabilities. Although evidence is currently limited to two studies with relatively small sample sizes, both studies point to a higher prevalence of PMDD in autistic women.

If increased PMDD in autism is associated with increased hormonal fluctuations (as argued by Obaydi & Puri, 2008), one can expect that also more severe complaints are experienced during menopause. The menopausal transition is characterized by large fluctuations in hormones (perimenopause), until after 12 months without menstruation post-menopause sets in. In this transitional phase, women experience many physiological changes which cause a wide variety of physical, psychological, sexual, and social problems (Nelson, 2008). Two qualitative studies investigating autistic women’s experience of the menopausal transition indicate that there are reasons to believe that this is a particularly difficult life phase, associated with large unmet (health) needs (Moseley et al., 2020, 2021). Unfortunately, due to the qualitative nature of these studies, and the lack of a comparison group, no inferences can be made whether this is specific for autistic women.

Next to diverging and/or more severe symptoms typically associated with hormonal changes, there are reports that autistic characteristics (such sensory differences and difficulties with regulating behaviors) are increased during menstruation (Steward et al., 2018), and menopause (Moseley et al., 2020, 2021) This heightening of autistic characteristics during menopause might cause an increase in autistic diagnoses during menopause in women. Moreover, in non-autistic women, menopausal symptoms are often associated with higher rates of psychiatric/psychological symptoms (Nelson, 2008). It is unclear whether the menopausal transition affects psychiatric/psychological symptoms differently in those already at a higher risk of psychiatric/psychological symptoms (i.e. women with ASC). Therefore, we will explore the relation between menopausal symptoms and psychological symptoms. We will focus on two common psychiatric conditions experienced by autistic people (anxiety and depression, see (Lever & Geurts, 2016)). As attention deficit hyperactivity disorder (ADHD) is a common neurodevelopmental comorbidity and one that was previously highlighted as potentially affected by the menopausal transition (Moseley et al., 2021), we also examined relationships between menopausal symptoms and ADHD symptomatology.

This preregistered study (AsPredicted #49299, https://aspredicted.org/ss5bm.pdf) aims to (a) replicate the findings of Lever and Geurts (2016) and Obaydi and Puri (2008), showing a higher prevalence of PMDD in autistic women, and we hypothesize that autistic women show an increased prevalence of PMDD; (b) explore whether autistic women also experience more/different menopausal symptoms during the menopausal transition, and (c) explore the relation between menopausal symptoms and symptoms of ASC, anxiety, depression, and ADHD.

## Method

### Participants

Participants were recruited via several mental health institutions (i.e. YOUZ (Leo Kannerhuis and SARR), GGZ Breburg, GGZe, Mondriaan, PsyQ, GGZ inGeest) across the Netherlands, (social) media (Twitter, LinkedIn, and Facebook), advertisements of autism networks (Dutch Association for autism www.nva.nl, Persons on the autism spectrum www.pasnederland.nl, Impuls https://impulsenwoortblind.nl/), and the social network of the researchers, research assistants, and students. The current study uses data of those participants that were newly recruited during Wave 3 of an ongoing study; also see Geurts et al. (2021). The following exclusion criteria were applied to all participants in the current study: (a) intellectual disability and/or Intelligence Quotient (IQ)-score <70 (based on two subtests of the WAIS-IV (Wechsler 2008), (b) insufficient understanding of Dutch language, (c) a history of neurological disorders (e.g. epilepsy, stroke, and multiple sclerosis), schizophrenia or having experienced more than one psychotic episode, and (d) current alcohol or drugs dependency. For the comparison group additional exclusion criteria were (a) one or more psychotic episodes, (b) a present or past diagnosis of an ASC, or a total score higher than 32 on the AQ or ASC diagnosis in close family members (i.e. parent(s), child(ren), brother(s), sister(s)), and (c) present/past diagnosis of ADHD or a total score of six of higher on the ADHD-SR, or a diagnosis of ADHD in close family members. Furthermore, participants in the ASC group were required to have a clinical *Diagnostic and Statistical Manual of Mental Disorders* (4th ed., DSM-IV), or DSM-5 diagnosis of an ASC.

The current study is part of a multicohort longitudinal study and follows participants that were already included in Lever and Geurts (2016). Samples that were used to address the different aims were partly overlapping. For our first aim (i.e. replicate findings by Lever & Geurts on PMDD), we included all participants (*n* = 70, *n*ASC = 28, *n*comparisons = 42) that were not already part of their original sample and were newly recruited during in a new wave of data collection (Wave 3). For analyses concerning menopausal complaints, we included all participants older than 40 (*n* = 65, ASC *n* = 30, comparisons *n* = 35) with self-reported irregular or absent menstruation patterns.

### Materials

PMDD (yes/no) was taken from the Mini international neuropsychiatric interview Plus (MINI-Plus; Sheehan et al., 1997; van Vliet et al., 2000). This is a structured diagnostic interview that explores several psychiatric disorders. We used lifetime prevalence, and this was assessed, retrospectively, in peri/postmenopausal women.

Menopausal complaints were examined using the Dutch version of the Menopause Rating Scale (MRS; Heinemann et al., 2003; Schneider et al., 2000). Total score, and three subscale scores, that is, psychological symptoms such as depression and anxiety (0–16); somato-vegetative symptoms, including hot flashes and cardiac complaints (0–16), and urogenital symptoms such as sexual complaints and vaginal dryness (0–12) were used. The MRS is sufficiently valid and reliable: the internal consistency coefficient measured with Cronbach’s alpha is 0.86.

Psychiatric/psychological symptoms were measured by subscale scores of the symptom checklist (SCL-90; Arrindell & Ettema, 2005; Derogatis, 1977) for depression, and the sum of anxiety and agoraphobic anxiety as a proxy of anxiety. The measure is considered valid, and the subscale as reliable with a Cronbach’s alpha of 0.89 (Hafkenscheid, 1993).

Furthermore, we used the ADHD-self-report (ADHD-SR; Kooij et al., 2005) to assess current ADHD symptoms, subdivided in inattention and hyperactive/impulsive symptoms. The instrument shows internal consistency and can be seen as valid (Döpfner et al., 2006).

The total score on the Autism Quotient (AQ) was used to assess autistic characteristics (Baron-Cohen et al., 2001; Hoekstra et al., 2008). The Dutch version of the AQ is sufficiently valid and reliable: the internal consistency coefficient measured with Cronbach’s alpha is 0.71

### Procedure

Written informed consent was obtained from all participants. The study was approved by the ethical review board of the Department of Psychology of the University of Amsterdam (2018-BC-9285). The current study was performed conforming to the principles of the Declaration of Helsinki Declaration of 1975, as revised in 2008. Participants underwent extensive testing protocol (for a full description of all procedures, see Geurts et al., 2021). Data used in the current study were obtained during Wave 3 of a longitudinal overlapping cohort study and concerning PMDD-only data were used of participants that were newly recruited during Wave 3. Participants received compensation for travel and a small reward (€17.50) for participation.

### Community involvement

A stakeholder group consisting of four autistic older adults (2 men, 2 women) is involved in all major aspects of the current research. The group meets 3 or 4 times a year, and during this meeting topics like study design, materials, results, and dissemination of results are discussed. The stakeholders receive compensation for their participation. For the current work, the setup and relevance of the study was discussed with the stakeholder group. They acknowledged the relevance of the current question and we discussed aspects of the menstrual cycle and/or menopause that they struggled with.

### Data analyses

All frequentist analyses were performed in R (RStudio Team, 2015). Age, IQ, mini mental state examination (MMSE), AQ, depression, anxiety and ADHD symptoms, and differences among groups were examined using a *t-*test. For education, a χ^2^ test was used. To test our first hypothesis, whether autistic women experience lifetime PMDD more often than non-autistic women, a χ^2^ test was used. Next, our second aim, whether the number of menopausal complaints differ between groups, was tested using a *t*-test with group (ASC/COMP) as the independent variable and MRS total score and subscales as the outcome. Bayesian analyses with default priors were performed in JASP (JASP Team, 2020) to assess the group effects. BF_10_ expresses the probability of the data given H1 relative to H0, BF_01_ expresses the probability of the data given H0 relative to H1 (please note that BF_01_ = 1/BF_10_). Bayes factors larger than 3 can be interpreted as substantial or stronger evidence for H1 (Lee & Wagenmakers, 2014).

To assess whether MRS scores are associated with psychological/psychiatric symptoms (depression, anxiety, and ADHD symptoms), regression analyses were performed. Separate models for the separate subscales of the MRS were run and for the separate psychiatric symptoms. Moreover, we explored whether relations differ between groups by adding the interaction with group to our models. In addition to our preregistration, we assessed whether autistic traits are associated with menopausal complaints. Correction for multiple comparisons took place using the Benjamini–Hochberg correction for four tests for all analyses with MRS as outcome.

## Results

### Participant characteristics

For our analyses for PMDD, information was available for 28 autistic women and 42 non-autistic women. The autistic women had more autistic traits, but groups did not differ in IQ, age, education, or MMSE score (also see Table 1, upper panel).

**Table 1.** Participant characteristics.

PMDD						
	Autism (*n* = 28)		Comparisons (*n* = 42)		*t*	*p* value
	*M* (*SD*)	Min–max	*M* (*SD*)	Min–max		
Age	49.8 (14)	31–72	56.2 (15.4)	31–79	−1.80	0.08
Gender (F/M/O)	28/0/0		42/0/0			
AQ	37.7 (6.7)	22–48	12.3 (5)	3–27	17.21	<0.001
IQ	114.7 (15.4)	85–144	111.6 (18)	73–137	0.78	0.44
MMSE	29.5 (0.6)	28–30	29.2 (0.9)	27–30	1.71	0.09
Education^a^	1/0/0/0/5/10/15		0/0/0/0/6/21/15			0.33
Menopause						
	Autism (*n* = 30)		Comparisons (*n* = 35)		*t*	*p* value
	*M* (*SD*)	Min–max	*M* (*SD*)	Min–max		
Age	58.5 (8.8)	42–73	62.9 (9.2)	45–79	−1.95	0.06
Gender (F/M/O)	30/0/0		35/0/0			
AQ	35.1 (6.6)	24–48	12.3 (5.3)	3–27	15.14	<0.001
IQ	116.8 (14.6)	92–144	119.1 (13.3)	85–137	−0.66	0.51
MMSE	29.2 (0.9)	27–30	29.2 (0.7)	28–30	−0.29	0.78
MRS total	14.1 (6.8)	3–33	6.42 (4.3)	0–17	5.28	<0.001
• Somatic	6.0 (2.7)	2–13	2.8 (2.1)	0–8	5.26	<0.001
• Psychological	5.2 (3.1)	0–10	1.7 (2.0)	0–7	5.28	<0.001
• Urogenital	2.7 (3.2)	0–10	1.7 (2.0)	0–9	1.43	0.16
Age at last menstruation	49.2(5.8)	38–60	50.1 (7.7)	36–59	−0.47	0.64
Education^a^	1/0/0/1/6/12/10		0/0/0/0/6/18/11			0.56

PMDD: premenstrual dysphoric disorder; F/M/O: female/male/other; AQ: autism quotient; IQ: intelligence quotient; MMSE: mini mental state examination; MRS: menopause rating scale.

a Education ranges from 1 (primary education not finished) to 7 (university level degree based on the Verhage coding system (1964)). For statistical testing, education levels were merged to prevent empty cells.

For analyses concerning menopausal complaints, data were available for 30 autistic women and 35 non-autistic women. Again, autistic women had more autistic symptoms, but groups did not differ in IQ, education, age, or MMSE score (also see Table 1, lower panel).

### PMDD

Lifetime PMDD was reported by 14.3% of autistic women and 9.5% of non-autistic women. This difference was not statistically significant, χ^2^(1) = 0.38, *p* = 0.54, and this lack of group differences was supported by Bayesian statistics (BF_10_ = 0.33, Supplementary Table S1).

### Menopausal complaints

**Figure 1.**
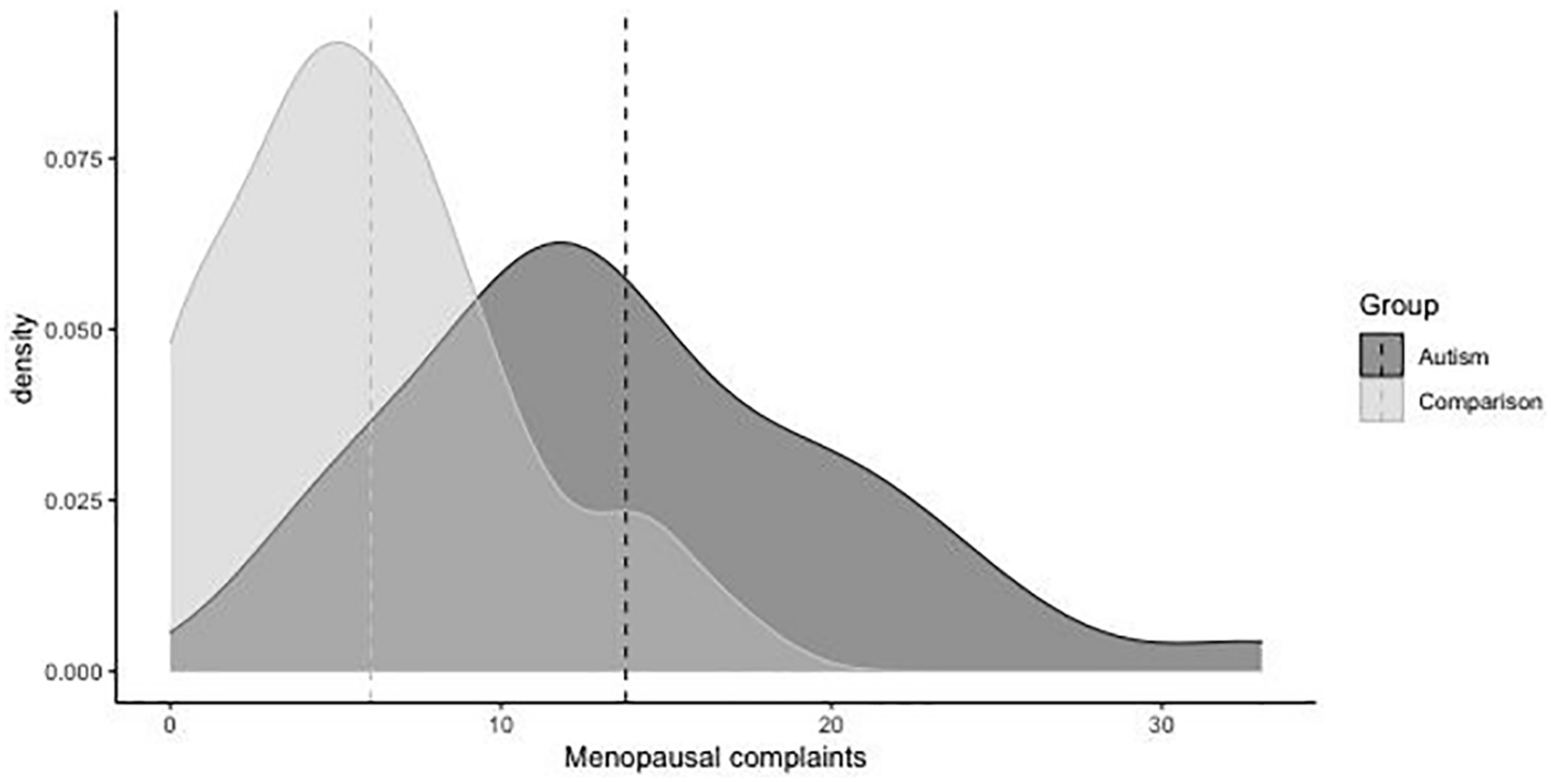
Density plot of total menopausal complaints. Dotted vertical lines indicate group means.

Autistic individuals had higher total menopausal complaints than non-autistic individuals (Figure 1). This translated into higher scores on subscales of psychological complaints, and somatic complaints, but not for urogenital symptoms (Table 1, lower panel). These results are supported by Bayesian statistics, except for urogenital symptoms there was no evidence for either hypotheses (see Supplementary Table S1).

**Figure 2.**
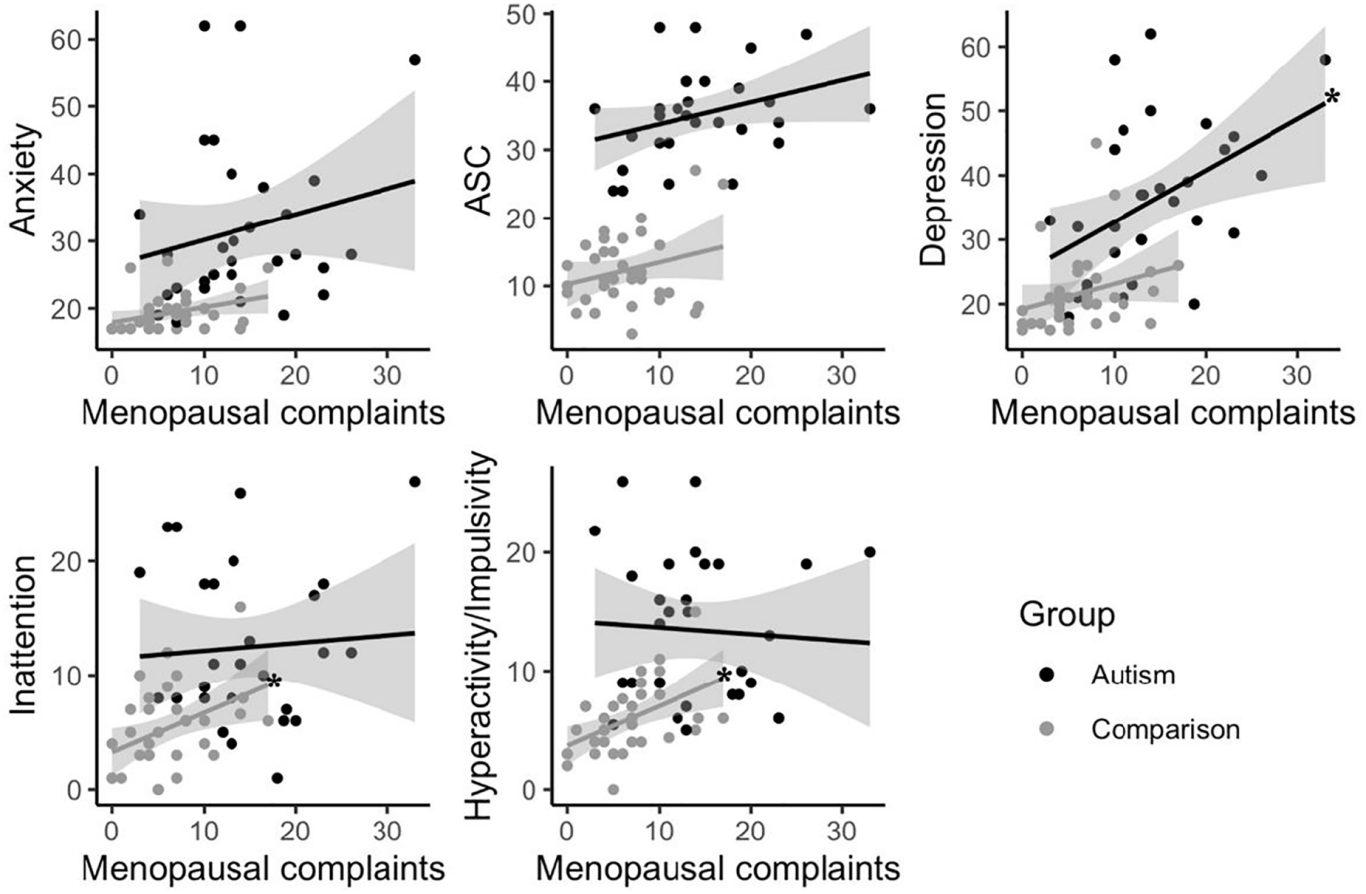
Relations between total menopausal complaints and psychiatric symptoms. Stars indicate significant relationships at *p* < 0.01.

As autistic individuals also scored higher on measures of anxiety, depression, autistic characteristics, inattention, and hyperactivity impulsivity, regression analyses were performed separately for autistic and non-autistic individuals. Total menopausal complaints were related to depressive symptoms in autism, but not in comparisons. In non-autistic women, significant associations were observed between total menopausal complaints and symptoms of inattention and hyperactivity impulsivity. No other significant associations were found between total menopausal complaints and psychological/psychiatric complaints (also see Figure 2).

Psychological menopausal complaints were associated with depressive symptoms in both groups, and with autistic characteristics in autistic individuals (Table 2). One autistic individual had extreme menopausal complaints (total score of 33). The association between total menopausal complaints and depression scores in autism was dependent on this participant and was no longer significant and smaller (*r* = 0.09) after removal (Supplementary Table S2).

**Table 2.** Relation between psychological symptoms and menopausal symptoms.

ASC	Menopausal complaints							
	Total		Psychological		Somatic		Urogenital	
	*r*	*p*	*r*	*p*	*r*	*p*	*r*	*p*
Anxiety	0.16	0.25	0.13	0.05	0.01	0.53	0.00	0.79
Depression	**0.26**	**0.01**	**0.31**	**0.001**	0.03	0.38	0.07	0.16
Total AQ	0.09	0.07	**0.21**	**0.01**	0.01	0.70	0.04	0.32
ADHD- IN	0.15	0.73	0.00	0.83	0.00	0.71	0.00	0.93
ADHD- H/I	0.15	0.75	0.03	0.32	0.01	0.52	0.06	0.20
Comparisons	Menopausal complaints							
	Total		Psychological		Somatic		Urogenital	
	*r*	*p*	*r*	*p*	*r*	*p*	*r*	*p*
Anxiety	0.12	0.04	0.16	0.02*	0.00	0.99	0.00	0.87
Depression	0.07	0.12	**0.26**	**0.002**	0.06	0.15	0.00	0.99
Total AQ	0.07	0.14	0.09	0.08	0.06	0.14	0.00	0.98
ADHD- IN	**0.17**	**0.01**	0.15	0.02*	0.02	0.46	0.06	0.15
ADHD- H/I	**0.24**	**0.003**	0.15	0.02*	0.08	0.10	0.09	0.08

ADHD: Attention-deficit/ hyperactivity disorder; AQ: autism quotient; ASC: autism spectrum conditions; H/I: hyperactivity impulsivity; IN: inattention.

Bolded figures are significant after correction for multiple comparisons.

* Not significant after multiple testing correction.

## Discussion

In autism research, men have been overrepresented for decades (Loomes et al., 2017), leading to a paucity of knowledge about common events in the lives of autistic women, such as menstruation and menopause. With this study we aimed to investigate the prevalence of PMDD and menopausal complaints in autistic women. In contrast to our hypothesis and previous studies (Lever & Geurts, 2016; Obaydi & Puri, 2008), we did not find that autistic women suffer from PMDD more often than non-autistic women. However, as hypothesized we found that autistic women do experience more menopausal complaints. Moreover, we explored whether these menopausal complaints were associated with common psychological/psychiatric complaints in both autistic and non-autistic women. Confirming our hypothesis, we found associations between menopausal complaints and psychological/psychiatric complaints in both autistic and non-autistic women.

We did not replicate the findings (Lever & Geurts, 2016; Obaydi & Puri, 2008) of increased lifetime prevalence of PMDD in autistic compared with non-autistic women. In our sample, autistic women reported a somewhat higher prevalence of PMDD (i.e. 14.5%) compared with previous studies in community samples of non-autistic women (around 8%; Dennerstein et al., 2012; Wittchen et al., 2002; Yonkers & Simoni, 2018). Autistic women in the current sample reported a lower prevalence of PMDD compared with previous studies on PMDD (Lever & Geurts, 2016 (21%) Obaydi & Puri, 2008 (92%)). As reported, prevalence rates of PMDD in autistic women vary so immensely between studies (14.5%–92% of autistic women) that studies in larger samples with more fine-grained measures are needed to elucidate specific causes and subgroups of women at risk.

The present study is the first to quantify menopausal complaints in autistic women. In agreement with previous qualitative studies (Moseley et al., 2020, 2021), we found that autistic women experience more complaints typically associated with menopause. Autistic women reported higher levels of total complaints, which was related to higher psychological and somatic menopausal complaints compared with non-autistic women, but not to higher urogenital complaints. Menopausal complaints are thought to be caused by highly irregular fluctuations in estrogens (Nelson, 2008). Previous studies have suggested that the hormonal balance is different in autistic women (e.g. Gasser et al., 2020; Ingudomnukul et al., 2007; Pohl et al., 2014), which could cause the increase in menopausal complaints in autistic women. However, future studies are necessary that look at the link between menopausal complaints, and estrogen levels in autistic women. A different, but likely complementary, explanation is that autistic women are more sensitive to changes in their body that occur during (peri) menopause due to an overall increased sensory sensitivity (Acevedo et al., 2018; Schauder & Bennetto, 2016). This may also possibly suggest that autistic women may experience menopausal complaints sooner than non-autistic women and might experience them for a longer period of time. Interestingly, also in ADHD increased menopausal complaints are noted (Dorani et al., 2021), suggesting that perhaps neurodiversity and its underlying etiology is also associated with increased difficulties during this transitional period. This could indicate that, for example, the interplay between neurotransmitters and sex (e.g. see Frey & Dias, 2014) might also play a part in the relation between neurodiversity and menopausal complaints. The interplay between hormonal levels, neurodiversity, and sensory sensitivity in the experience of menopause is currently unknown. While there is an obvious need for such studies, the methods used for the measurement of estrogen levels lack accuracy and precision when concentrations are lower as in menopause (Rosner et al., 2013). Careful thought and consideration have to be put into designing such studies.

Social and societal explanations for the experience of complaints typically associated with menopause in autistic women should also be considered. A lower impact of the menopausal transition is associated with stronger social support during stressful life events (Arnot et al., 2021), greater physical fitness, better coping strategies, and better sleep quality (Duffy et al., 2013). All these factors are generally rated lower in those with an ASC (Bishop-Fitzpatrick & Rubenstein, 2019; Ee et al., 2019; Hirvikoski & Blomqvist, 2015; Maxwell-Horn & Malow, 2017), suggesting that these could also impact the experience of menopause in autistic individuals. Moreover, autistic individuals might have more difficulty expressing their experience of menopause, and thus have more difficulties articulating their need for support (Moseley et al., 2021). Most likely, biological and nonbiological factors contribute to the experience of menopause. The interplay between these is possibly interactive, where some factors reinforce, and others attenuate each other. Therefore, future research should investigate these in a multidisciplinary way to unravel which factors contribute to the experience of the menopausal transition in autistic individuals.

Menopause is often associated with increased levels of depression and anxiety. Indeed, next to increases in psychological complaints related to menopause, our results also indicate that increased psychological menopausal complaints were associated with depressive symptoms, although in ASC this effect was driven by a single individual with extreme scores. More surprising is the positive association between characteristics of ADHD (inattention and hyperactivity/impulsivity) and menopausal complaints in non-autistic women, which we did not detect in autistic women. Moreover, we also found an increase in autistic characteristics associated with psychological menopausal complaints in autistic women. Clinically, this finding of increased psychological complaints during menopause is of major significance, as women might seek help for these problems. Moreover, characteristics of ASC or ADHD in this period might require a different clinical approach. Especially the relationship between menopausal complaints and ASC and ADHD characteristics is of interest, as we do not know whether or not these characteristics are transient (i.e. disappear after menopause). Knowing whether these are transient or not will impact both ASC and ADHD assessment during this hormonal phase. Moreover, we do not know whether evidence-based interventions will be equally effective during menopause. The relationship of ASC characteristics with menopausal complaints in autistic women specifically fits well with the idea that autistic women could be able to mask their autistic characteristics until they reach menopause, as also indicated in Moseley et al. (2020).

The current study is the first to quantitatively assess menopausal complaints in ASC. However, the results should be seen in the light of some limitations. We did not have any information about hormone therapy, often used to control symptoms of menopause (Steward et al., 2018). If either autistic or non-autistic women have less access to such treatments, this could have biased our results. Second, menopausal complaints change considerably over time. Unfortunately, we did not have the opportunity to investigate such patterns, and whether such patterns are different in autistic and non-autistic women. To accurately perform such a study, one would need large and importantly longitudinal samples. Moreover, menopause is notoriously difficult to accurately determine, and the current report relies on self-report of menopausal complaints, making it unclear whether the women in our sample are actually going through menopause. As we currently do not know whether autistic women might always experience more symptoms typically associated with menopause, whether autistic women might experience their last period at a different age, or whether menopause might last longer or shorter in autistic women, relying on normative tools is a limitation of the current study. Fourth, our models concerning psychological characteristics do not take causal relations between symptoms (e.g. impulsively interrupting others, might cause social anxiety in situations where this impulsivity might occur) and/or overlap (e.g. inattention in ADHD, might be similarly rated as attention problems that occur in depression) between the predictors (anxiety, depression, and ADHD traits) into account. To unravel the first possibility, longitudinal studies with multiple measurement times are necessary. The latter can be taken into account by using more sophisticated statistical models (e.g. using LASSO regression to determine the strongest predictor), but these need higher power than we had in the current study. Moreover, it is likely that both causal relations between psychological characteristics and overlap between psychological characteristics exist. To unravel these, more research is warranted. Finally, we used the AQ to quantify autistic characteristics. Previous studies have emphasized the male biases in ASC measures (Lai et al., 2015), and that women might meet diagnostic criteria in a different manner than men do.

In conclusion, we show increased menopausal complaints in autistic women. Moreover, we found that menopausal complaints were associated with increased levels of psychological/psychiatric symptoms. In contrast with previous work, we did not find an increased prevalence of PMDD in autistic women. With this work, we show the important role that major reproductive milestones can have in an autistic woman’s life. Increasing knowledge about the causes and impact of hormonal changes is of major importance, to provide women with the support that they require in every life phase.

## Supplemental Material

sj-docx-1-aut-10.1177_13623613211059721 – Supplemental material for Menstruation and menopause in autistic adults: Periods of importance? Click here for additional data file. Supplemental material, sj-docx-1-aut-10.1177_13623613211059721 for Menstruation and menopause in autistic adults: Periods of importance? by Annabeth P Groenman, Carolien Torenvliet, Tulsi A Radhoe, Joost A Agelink van Rentergem and Hilde M Geurts in Autism

## Notes

1. In the current manuscript we include every individual who menstruates, regardless of gender. As all individuals included in this manuscript identified as cisgender, we will continue to use “women”. Sex is the label you are assigned at birth, related to a person’s biology, anatomy and chromosomes, while gender is based on social and cultural expectations, norms, views and behaviors associated with a specific sex. Importantly, sex and gender are often used interchangeably, however even though both can influence research results, they represent a different concept.

2. We identify use of first language (also see Kenny et al., 2016) throughout this article. However, we want to note the lively discussion about person-first, versus identity-first language.
