# Supplementary material for: Menstruation and menopause in autistic adults: Periods of importance?
Source: Autism. 2021 Nov 26;26(6):1563–72. doi: 10.1177/13623613211059721 (PMC9344571; doi:10.1177/13623613211059721)
Supplement: sj-docx-1-aut-10.1177_13623613211059721 – Supplemental material for Menstruation and menopause in autistic adults: Periods of importance? [file sj-docx-1-aut-10.1177_13623613211059721.docx]

Table S1. Bayesian statistics for group outcomes

|  | PMDD | Total menopausal complaints | Psychological menopausal complaints | Somatic menopausal complaints | Urogenital complaints |
| --- | --- | --- | --- | --- | --- |
| BF10 | .33 | 13514.06 | 14763.85 | 11098.12 | 1.56 |
| BF01 | 3 | <.001 | <.001 | <.001 | .64 |

Table S2. MRS outcomes no outlier

| Autism | | | | | | |  | |
| --- | --- | --- | --- | --- | --- | --- | --- | --- |
|  | Total menopausal complaints | | Psychological menopausal complaints | | Somatic menopausal complaints | | Urogenital complaints | |
|  | r_adj_ | *p* | r_adj_ | *p* | r_adj_ | *p* | r_adj_ | *p* |
| Anxiety | -.04 | .95 | .06 | .10 | -.03 | .64 | .00 | .30 |
| Depression | .09 | .07 | **.24** | **.003** | -.04 | .99 | -.01 | .37 |
| Total AQ | .11 | .05 | **.19** | **.01** | -.03 | .72 | .00 | .33 |
| ADHD- IN | .00 | .35 | -.03 | .76 | -.01 | .42 | -.01 | .40 |
| ADHD- H/I | .00 | .31 | -.01 | .45 | .03 | .18 | .07 | .08 |
| Comparisons | | | | | | |  | |
|  | Total menopausal complaints | | Psychological menopausal complaints | | Somatic menopausal complaints | | Urogenital complaints | |
|  | r_adj_ | *p* | r_adj_ | *p* | r_adj_ | *p* | r_adj_ | *p* |
| Anxiety | .09 | .04 | .13 | .02* | -.03 | .99 | -.03 | .87 |
| Depression | .05 | .12 | **.24** | **.002** | .03 | .15 | -.03 | .99 |
| Total AQ | .04 | .14 | .06 | .08 | .04 | .14 | -.03 | .98 |
| ADHD- IN | **.15** | **.01** | .13 | .02* | -.01 | .46 | .03 | .15 |
| ADHD- H/I | **.22** | **.003** | .12 | .02* | .05 | .10 | .06 | .08 |
